# Repeating Physicians Prescriptions

**Published:** 1877-12

**Authors:** 


Repeating Physicians' Prescriptions.—Physicians complain that their prescriptions are not only used by their patients, but handed round oftentimes amongst a large circle of friends, and in many instances handed down from generation to generation as a kind of heirloom. This custom has its attendant dangers, and in the case of poisons might be productive of bad results, to remedy which it is proposed in Germany to institute a law prohibiting chemists to make up any prescriptions containing strong remedies—as drastics, emmenagogues, sedatives, etc.—unless the prescription is again countersigned by the medical man who originally gave it.—*Medical Press and Circ.*

